# Long Non-coding RNA GAS5 Maintains Insulin Secretion by Regulating Multiple miRNAs in INS-1 832/13 Cells

**DOI:** 10.3389/fmolb.2020.559267

**Published:** 2020-09-24

**Authors:** Yanwei Luo, Jie Guo, Pingsheng Xu, Rong Gui

**Affiliations:** ^1^Department of Blood Transfusion, The Third Xiangya Hospital of Central South University, Changsha, China; ^2^National Institution of Drug Clinical Trial, Xiangya Hospital, Central South University, Changsha, China

**Keywords:** gene therapy, GAS5, insulin secretion, long non-coding RNA, type-2 diabetes

## Abstract

Type-2 diabetes mellitus (T2DM) is a complex disease characterized by reduced pancreatic islets β-cell mass and impaired insulin release from these cells. Non-coding RNAs, including microRNAs (miRNA) and long non-coding RNAs (lncRNAs), play a role in the progression of T2DM. Decreased serum lncRNA GAS5 levels were reported to be associated with T2DM. However, the role of GAS5 in regulating islet cell functions remain unknown. In this study, we found that the serum levels of GAS5 were significantly lower in patients with T2DM compared with healthy control subjects, and the low serum GAS5 levels were associated with high levels of HbAlc and fasting glucose in patients with T2DM. In addition, we found that serum GAS5 levels were negatively correlated with the serum levels of miR-29a-3p, miR-96-3p, and miR-208a-3p in patients with T2DM. Consequently, using INS-1 832/13 rat β-cell line, we found that overexpression of GAS5 by lentivirus infection increased glucose-stimulated insulin secretion and insulin content compared with negative control, whereas knockdown of GAS5 expression reduced both them. Moreover, GAS5 interacted with miR-29a-3p, miR-96-3p, and miR-208a-3p in INS-1 832/13 cells, as judged by pull-down assay and dual luciferase reporter assay. GAS5 overexpression caused the decrease in expression of miR-29a-3p, miR-96-3p, and miR-208a-3p in INS-1 832/13 cells and conversely caused the increase in expression of insulin receptor, insulin receptor substrate, and phosphoinositide-3-kinase regulatory subunit 1. Thus, these results reveal a novel mechanism whereby GAS5 is involved in maintaining insulin secretion and may represent a novel therapeutic target for T2DM.

## Introduction

Type-2 diabetes mellitus (T2DM) is a complex disease characterized by diminishing pancreatic islet β-cell mass and impaired insulin release from these cells (Wessel et al., 2015). T2DM has become one of the leading causes of morbidity and mortality and costs astronomical sums of money in annual healthcare in worldwide. Non-coding RNAs, including microRNAs (miRNAs) and long non-coding RNAs (lncRNAs) have been reported to play a role in the progression of T2DM (Chang, 2017). These non-coding RNAs are crucial for controlling the expression of genes related to insulin release, which points to unknown regulatory network governing islet cell functions (Akerman et al., 2017). LncRNAs function as biomarkers in the personalized treatment of T2DM (Lin et al., 2019; Liu et al., 2019). Researchers have discovered thousands of lncRNAs that highly enriched or specifically expressed in pancreatic islet β-cells (Qi et al., 2017). Carter et al. (2015) demonstrated that decreased serum LncRNA GAS5 (growth arrest-specific transcript 5) levels were associated with higher risk for having T2DM (Carter et al., 2015). GAS5 is extensively expressed in various tissues and organs and plays a key role in cell apoptosis and growth. Jin et al. found that the GAS5 level was significantly decreased in db/db mice. Knockdown of GAS5 expression led to cell cycle G1 arrest and impaired insulin synthesis and secretion in Min6 cells (Jin et al., 2017). A recent study demonstrates that modulation of GAS5 in the human beta cell alleviated the glucocorticoid-induced insulin secretion defect, suggesting the potential of GAS5 as a new regulator, maintaining β cell identity and function by affecting insulin synthesis and secretion (Esguerra et al., 2020).

However, the underlying mechanisms how GAS5 regulates islet cell functions are not fully clear. LncRNA, as a competitive endogenous RNA (ceRNA), binds to miRNA and acts as a miRNA sponge, thereby reducing the activity of miRNA and indirectly up-regulating the expression of miRNA-related target genes (Goustin et al., 2019). It has been reported that GAS5 targets to miR-452-5p and miR-221-3p in renal tubular cells and mesangial cells, which are involved in diabetic nephropathy (Ge et al., 2019; Xie et al., 2019).

This study aimed to identify the potential mechanism of GAS5 as a miRNA sponge in maintaining islet cell function. We demonstrated that serum GAS5 level was downregulated in patients with T2DM compared with healthy controls and was associated with high levels of HbAlc and fasting glucose. Overexpression of GAS5 by lentivirus infection increased glucose-stimulated insulin secretion and insulin content. By the bioinformatics analysis, pull-down and luciferase assay, we revealed that GAS5 interacted with miR-29a-3p, miR-96-3p, and miR-208a-3p, thereby regulating the expression of insulin receptor (INSR), insulin receptor substrate 1 (IRS-1), and phosphoinositide-3-kinase regulatory subunit 1 (PIK3R1) in INS-1 832/13 cells. Therefore, GAS5 might serve as a regulator in pancreatic β cell function and T2DM development through acting as a miRNA sponge.

## Materials and Methods

### Tissue Samples

One hundred and twenty-two serum samples from health people and 88 serum samples from type-2 diabetes were collected at Xiangya Hospital, Central South University (Changsha, China). The statistical number required for this study was estimated by G^∗^Power software (Faul et al., 2007). According to the WHO diagnostic criteria in 1999 and oral glucose tolerance test (OGTT), the patients with fasting blood glucose (FPG) ≥7.0 mmol/L and 2 h postprandial blood glucose (2 h PG) ≥ 11.1 mmol/L were included in this study. Exclusion criteria: the patients with hypertension, malignant tumors, lung diseases, polycystic ovary syndrome, autoimmune diseases; patients with other diseases affecting glucose metabolism, such as hyperthyroidism, acromegaly, and Cushing’s syndrome, acute metabolic abnormalities syndrome or other stress conditions and smokers were excluded. The subjects with fasting blood glucose (FPG) <6.1 mmol/L and 2 h postprandial blood glucose (2 h PG) <7.8 mmol/L were included as healthy controls in this study. The basic clinical information of 122 healthy subjects and 88 patients with T2DM was shown in Table 1. The samples were snap-frozen in liquid nitrogen, and then stored at −80°C for further use. This project was approved by the Ethic Committee of Xiangya Hospital, Central South University. All subjects have signed the informed consent.

**TABLE 1:** Characteristics of subjects in this study.

	Healthy (*n* = 122)	T2DM (*n* = 88)
Sex, male/female	45/77	38/50
Age (years)	54 ± 4.8	56 ± 6.3
Glycated hemoglobin (%)	5.2 ± 0.2	9.4 ± 1.8*
Fasting plasma glucose (mmol/L)	4.7 ± 0.3	8.6 ± 1.3*
Total cholesterol (mmol/L)	4.3 ± 0.4	4.5 ± 0.4
Triglycerides (mmol/L)	0.9 ± 0.1	1.3 ± 0.2*
HDL-c (mmol/L)	1.3 ± 0.1	1.0 ± 0.2*
LDLc (mmol/L)	2.2 ± 0.3	3.5 ± 0.5*
History of hypertension (yes/no)	0/122	10/78
Coronary heart disease (yes/no)	0/122	7/81
Diabetic nephropathy (yes/no)	0/122	8/80
OHA, number (yes/no)	0/122	45/43
Insulin, number (yes/no)	0/122	37/51

*p < 0.05 compared with the healthy control. HDL-c: High density lipoprotein cholesterol; LDL-c: Low density lipoprotein cholesterol; OHA: oral hypoglycemic agents; T2DM: type 2 diabetes mellitus.

### Cell Culture

Rat β-cell line INS-1 832/13 and pancreatic β cell line Min6 were purchased from National infrastructure of Cell Line Resource (Beijing, China) and grown routinely in RPMI-1640 medium with 11.1 mM D-glucose (cat no. 984313, Invitrogen, CA, United States) supplemented with 10% fetal bovine serum (cat no. 16140071, Gibco, CA, United States) and 50 μmol/L 2-mercaptoethanol (cat no. 21985023, Invitrogen, CA, United States). Culture conditions were maintained at 37°C in a humidified atmosphere of 5% CO_2_. The cells were used for performing experiment after 4 times of passages.

### Over-Expression and Knock-Down of GAS5

To knock down GAS5, the shRNA sequence of GAS5 (shRNA 1# 5′-GCCTAACTCAAGCCATTGG-3′ or shRNA 2# 5′-GGTATGGAGAGTCGGCTTG-3′) was inserted into psi-LVRU6GP vector (GeneCopoeia, Guangzhou, China), which was packaged into lentivirus by GeneCopoeia Co., Ltd. (Guangzhou, China). The cells transfected with lentivirus containing scramble sequence (scramble sequence: 5′-GCGACACGCGCATCTATAT-3′) were used as a control for shRNA transfection. To overexpress GAS5, the sequence of rat GAS5 (based on the rat GAS5 sequence, NR_002704.1, 429 bp) was directly synthesized and inserted into pReceiver-Lv218 vector (GeneCopoeia, Guangzhou, China), which was packaged into lentivirus by GeneCopoeia Co., Ltd. (Guangzhou, China). The cells transfected with lentivirus carrying empty pReceiver-Lv218 vector were used as a control for overexpression transfection. Briefly, cells were plated in 6-well clusters or 96-well plates for 12 h and then infected with lentivirus at multiplicity of infection (MOI) = 50 for 48 h. Transfected cells were used in further assays or RNA/protein extraction.

### RNA Extraction and SYBR Green Quantitative PCR Analysis

The total RNA in serum samples of the patients and healthy controls was extracted using Total RNA Purification Kit (cat no. 37500, Norgen Biotek Corp., Thorold, ON, Canada) according to the manufacturer’s instructions. Total RNA in cells was extracted using TRIzol reagent (cat no. 15596018, Invitrogen, CA, United States). Mature miR-29a-3p, miR-96-3p, miR-208a-3p, miR-452-5p, and miR-221-3p in cells and serum samples were detected using a Hairpin-it miRNAs qPCR kit (cat no. E01009, GenePharma, Shanghai, China) according to manufacturers’ instructions. Expression of RNU6B was used as an endogenous control. The expression of GAS5, insulin receptor (INSR), insulin receptor substrate 1 (IRS-1), and phosphoinositide-3-kinase regulatory subunit 1 (PIK3R1) was measured by TB green qPCR assay (cat no. RR096A, Takara, Dalian, China) according to manufacturers’ instructions. Expression of β-actin was used as an endogenous control. We aligned the sequences of GAS5 and miRNAs between human and rat using Blast. The sequences have a high similarity between human and rat (92.59% for GAS5, 98.96% for miR-96-3p, 98.57% for miR-29a-3p, and 95.77% for miR-208a-3p). We designed the primers from the identical sequences between human and rat to perform qPCR. The primers were used as followings: GAS5, sense, 5′-TGTGTCCCCAAGGAAGGATG-3′, anti-sense, 5′-TCCACACAGTGTAGTCAAGCC-3′; INSR, sense, 5′-AG ACGTCCCGTCAAATATTGC-3′, anti-sense, 5′-ACTCGTTG ACCGTCTTCACC-3′; IRS-1, sense, 5′-GCGGTTTCATCT CCTCGGAT-3′, anti-sense, 5′-GGGTAGGCAGGCATCATCTC-3′; PIK3R1, sense, 5′-ACATTTTTCATGCATGTTTTCCAG-3′, anti-sense, 5′-ATGGGATAGTGCCTGAGCCT-3′; β-actin, sense, 5′-AGGGGCCGGACTCGTCATACT-3′, anti-sense, 5′-GGCGG CACCACCATGTACCCT-3′. QPCR was performed in ABI 7500 Real-Time PCR System (SeqGen, Inc., Torrance, CA, United States) at the condition: 95.0°C for 3 min, and 39 circles of 95.0°C for 10 s and 60°C for 30 s. Data were processed using 2^–Δ^ ^Δ^ ^*CT*^ method.

### Insulin Secretion Assay

Confluent plates of INS-1 832/13 cells were washed with 1 mL pre-warmed secretion assay buffer (SAB, 986.82 mM NaCl, 93.88 mM KCl, 118.02 mM MgSO4.7H_2_O,118.04 mM KH_2_PO_4_, 833.23 mM NaHCO_3_, 100 mM HEPES, and 83.93 mM CaCl_2_.2H_2_O containing 0.5% BSA). Cells were then pre-incubated in fresh 2 mL SAB with 2.8 mM glucose for 2 h. The cells were then stimulated with 2.8 mM glucose or 16 mM glucose in 1 mL SAB at 37°C for 1 h. The insulin levels were normalized to total protein from each well. Total protein from each well was extracted by using 200 μL RIPA buffer (cat no. 0278, Roche, NJ, United States). The protein content was measured by BCA assay (cat no. 23227, Pierce, IL, United States).

### Total Insulin Measurement

Cells transfected with lentivirus carrying GAS5 DNA or scramble DNA sequence as described above were lysed in 200 μL RIPA buffer after 72 h post-transfection. Total insulin was determined using Mercodia High Range Rat Insulin ELISA (cat no. 10-1145-01, Mercodia AB, Sweden), respectively, according to the manufacturer’s protocol.

### Western Blot Analysis

Immunoblotting was performed to detect the expression of INSR, IRS-1 and PIK3R1. Cultured cells or transfected cells were lysed in RIPA buffer with 1% PMSF. Protein was loaded onto an SDS-PAGE minigel and transferred onto PVDF membrane. After probed with primary antibody (anti-INSR (cat no. YT6040, 1:1000), IRS-1 (cat no. YT2410, 1:1000) and PIK3R1 (cat no. YM3408, 1:1000) from ImmunoWay Biotechnology Company, Plano, TX, United States; anti-GAPDH (cat no. ab8245, 1:2000) from Abcam, Cambridge, United States) at 4°C overnight, the blots were subsequently incubated with HRP-conjugated secondary antibody (cat no. SA009, 1:5000, Auragene, Changsha, China) for 1 h at 37°C. Signals were visualized using Immobilon ECL Ultra Western HRP Substrate (cat no. WBULS0500, Millipore, MA, United States). GAPDH was used as an endogenous protein for normalization.

### Luciferase Reporter Gene Assay

The wild type fragment of GAS5 (GAS5-wt) containing the putative binding site on miR-29a-3p, miR-96-3p, and miR-208a-3p was amplified by PCR. The PCR product was subcloned into a psiCHECK-2 vector (Promega, Madison, WI, United States) immediately downstream to the luciferase gene sequence. The mutant type binding site of GAS5 (GAS5-mut) with miR-29a-3p, miR-96-3p, and miR-208a-3p was synthesized by GenePharma Co. Ltd. (Shanghai, China). All constructs were verified by DNA sequencing. The cells were plated in 96-well clusters, and then transfected with 100 ng GAS5-wt or GAS5-mut construct with or without miR-29a-3p, miR-96-3p, and miR-208a-3p mimics (RiboBio Co. Ltd., Guangzhou, China). At 48 h after transfection, luciferase activity was detected using a dual-luciferase reporter assay system (Promega, Madison, WI, United States) and normalized to Renilla activity.

### MTT Assay

Each group of INS-1 832/13 (5 × 10^3^ each wells) was seeded into a 96-well plate fulfilled with 200 μl medium and continually cultured for 0, 24, 48, or 72 h. At the indicated end points, 20 μl of MTT solution (5 mg/ml, cat no. M6494, Invitrogen, CA, United States) was added to each well, and incubated for 4 h. And then 150 μl of dimethyl sulfoxide was added to each well to dissolve the crystals completely. The absorbance at 570 nm of each well was measured by a microplate reader.

### Pull-Down Assay With Biotinylated GAS5 Probe

The biotinylated GAS5 probe was synthesized by GenePharma Co. Ltd. (Shanghai, China). The probes were incubated with streptavidin-coated magnetic beads (Sigma) at room temperature for 4 h to generate probe-coated magnetic beads. INS-1 832/13 cells were lysed by RIPA buffer. The lysates were incubated with probe-coated beads at 4°C overnight. The next day, the beads were washed with wash buffer for three times. The RNA complexes bound to the beads were eluted and extracted by for quantitative PCR analysis.

### Statistical Analysis

All data from three independent experiments were expressed as mean ± S.D. and processed using SPSS17.0 statistical software. The difference among the groups was estimated by Student’s *t*-test or one-way ANOVA depending on the conditions. The correlation of GAS5 and miRNAs was analyzed by Spearman correlation analysis. The association of GAS5 and miRNAs with demographic and biochemical parameters of patients with type 2 diabetes mellitus was analyzed by chi-square test. A *P* value of <0.05 was statistically significant.

## Results

### The Levels of GAS5, miR-29a-3p, miR-96-3p, miR-208a-3p in Serum Samples From Patients With T2DM

**FIGURE 1:**
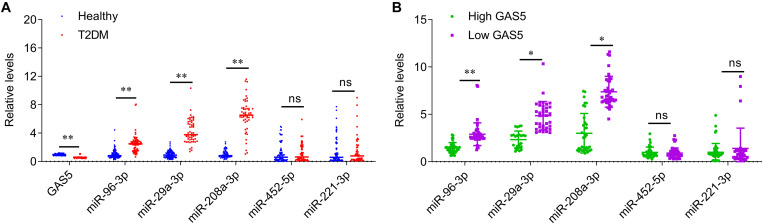
The serum levels of GAS5 and miRNAs in patients with type-2 diabetes and healthy control subjects. **(A)** QPCR was performed to determine the serum levels of GAS5, miR-29a-3p, miR-96-3p, miR-208a-3p, miR-452-5p, and miR-221-3p in patients with type-2 diabetes and healthy control subjects. **(B)** The serum levels of miRNAs in low GAS5 group and high GAS5 group according to the mean level of GAS5 in patients with type-2 diabetes. Data were presented as mean ± SD. T2DM, type-2 diabetes mellitus; ns, no significant. *p < 0.05, **p < 0.01 vs. healthy control.

**FIGURE 2:**
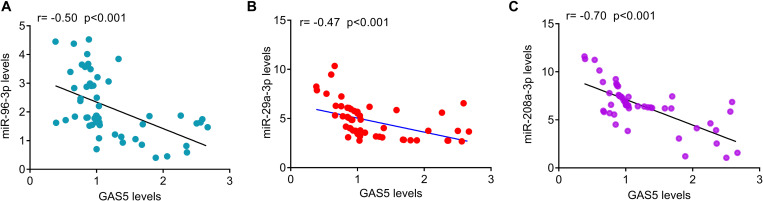
The correlation of serum GAS5 levels with serum miRNAs levels in patients with T2DM. **(A)** Serum GAS5 levels were negatively correlated with miR-96-3p levels in patients with type-2 diabetes (*r* = –0.50, *p* < 0.001). **(B)** Serum GAS5 levels were negatively correlated with miR-29a-3p levels in patients with type-2 diabetes (*r* = –0.47, *p* < 0.001). **(C)** Serum GAS5 levels were negatively correlated with miR-208a-3p levels in patients with type-2 diabetes (*r* = –0.70, *p* < 0.001). r: Spearman correlation coefficient.

The relative levels of GAS5 and miRNAs were measured by quantitative PCR analysis in serum samples from patients with T2DM and healthy controls. The results showed that the serum levels of GAS5 were significantly lower in patients with T2DM, while the serum levels of miR-29a-3p, miR-96-3p, miR-208a-3p were significantly higher in patients with T2DM compared with health control subjects (Figure 1A). By contrast, the serum levels of miR-452-5p and miR-221-3p were similar between the two groups (Figure 1A). In addition, we divided the patients into low GAS5 group (*n* = 58) and high GAS5 group (*n* = 30) according to the mean level of GAS5 (Tables 2, 3). The levels of miR-29a-3p, miR-96-3p, miR-208a-3p were significantly lower in high GAS5 group compared with low GAS5 group, while the levels of miR-452-5p and miR-221-3p were comparable between high GAS5 group and low GAS5 group (Figure 1B). Furthermore, we found that serum GAS5 levels were negatively correlated with serum levels of miR-96-3p (*r* = −0.50, *p* < 0.001), miR-29a-3p (*r* = −0.47, *p* < 0.001), and miR-208a-3p (*r* = −0.70, *p* < 0.001) (Figure 2). These results provide a first evidence suggesting that the decrease in GAS5 expression plays a key role in development of T2DM.

**TABLE 2:** The association of GAS5, miR-96-3p, miR-29a-3p, and miR-208a-3p with continuous variables of patients with T2DM.

Variables	GAS5			miR-96-3p			miR-29a-3p			miR-208a-3p		
	Low (*n* = 58)	High (*n* = 30)	*p*	Low (*n* = 40)	High (*n* = 48)	*p*	Low (*n* = 38)	High (*n* = 50)	*p*	Low (*n* = 36)	High (*n* = 52)	*p*
**Continuous variables**												
Age (mean @ SD)	55 ± 4.5	57 ± 7.5	0.121	55 ± 6.4	56 ± 3.2	0.345	56 ± 2.6	55 ± 5.2	0.281	57 ± 4.6	55 ± 6.2	0.103
HbAlc (%)	9.9 ± 0.8	8.5 ± 0.8	0.000*	8.8 ± 0.7	9.7 ± 0.6	0.000*	8.9 ± 0.8	9.6 ± 0.5	0.000*	8.7 ± 0.8	9.8 ± 0.8	0.000*
FG (mmol/L)	9.8 ± 0.7	7.9 ± 0.5	0.000*	8.1 ± 0.8	9.5 ± 0.7	0.000*	8.2 ± 1.2	9.5 ± 1.9	0.004*	8.2 ± 0.3	9.1 ± 0.5	0.000*
TC (mmol/L)	4.6 ± 0.9	4.4 ± 1.1	0.363	4.7 ± 1.0	4.4 ± 1.1	0.188	4.5 ± 0.7	4.6 ± 0.8	0.542	4.6 ± 0.4	4.5 ± 0.7	0.441
TG (mmol/L)	1.2 ± 0.6	1.4 ± 0.5	0.121	1.3 ± 0.6	1.3 ± 0.7	1.000	1.1 ± 0.4	1.4 ± 0.6	0.009*	1.0 ± 0.7	1.4 ± 0.5	0.002*
HDL-c (mmol/L)	0.9 ± 0.5	1.1 ± 0.6	0.101	1.0 ± 0.6	1.1 ± 0.4	0.354	1.2 ± 0.6	0.9 ± 0.8	0.056	1.0 ± 0.4	1.1 ± 0.7	0.441
LDL-c (mmol/L)	3.8 ± 0.9	3.4 ± 0.8	0.043*	3.7 ± 0.5	3.6 ± 0.7	0.451	3.2 ± 0.8	3.6 ± 0.6	0.009*	3.7 ± 0.3	3.4 ± 0.8	0.035*

*HbAlc: Glycated hemoglobin; FG: Fasting glucose; TC: Total cholesterol; TG: Triglycerides; HDL-c: High density lipoprotein cholesterol; LDL-c: Low density lipoprotein cholesterol; T2DM: type 2 diabetes mellitus. 0.000: *p* < 0.0001. ^∗^*p* < 0.05.*

**TABLE 3:** The association of GAS5, miR-96-3p, miR-29a-3p, and miR-208a-3p with categorical variables of patients with T2DM.

Variables	GAS5			miR-96-3p			miR-29a-3p			miR-208a-3p		
	Low (*n* = 58)	High (*n* = 30)	*p*	Low (*n* = 40)	High (*n* = 48)	*p*	Low (*n* = 38)	High (*n* = 50)	*p*	Low (*n* = 36)	High (*n* = 52)	*p*
**Categorical variables**												
Hypertension (N)			0.730			0.336			1.000			0.734
Yes	6	4		3	7		4	6		5	5	
No	52	26		37	41		34	44		31	47	
Coronary heart disease (N)			1.000			0.697			0.694			0.233
Yes	5	2		4	3		2	5		1	6	
No	53	28		36	45		36	45		35	46	
Diabetic nephropathy (N)									0.284			1.000
Yes	5	3	1.000	4	4	1.000	5	3		3	5	
No	53	27		36	44		33	47		33	47	
OHA, number (N)			0.000*			0.002*			0.138			0.054
Yes	18	27		28	17		23	22		23	22	
No	40	3		12	31		15	28		13	30	
Insulin, number (N)			0.000*			0.197			0.004*			0.048*
Yes	12	25		20	17		23	14		20	17	
No	46	5		20	31		15	36		16	35	

*OHA: oral hypoglycemic agents; T2DM: type 2 diabetes mellitus. 0.000: *p* < 0.0001. ^∗^*p* < 0.05.*

### The Association of GAS5 Levels With Biochemical Parameters in Patients With T2DM

We also analyzed the associations between GAS5 or miRNAs and various clinical parameters of the 88 patients with T2DM (Tables 2, 3). We found that the low serum levels of GAS5 were associated with high levels of HbAlc, fasting glucose, and LDL-c in patients with T2DM (Table 2). In addition, the percentage of patients with high serum GAS5 was higher in patients treated with oral hypoglycemic agents or insulin than in patients without the treatments (Table 3). By contrast, the high levels of miR-29a-3p, miR-96-3p, and miR-208a-3p were associated with high levels of HbAlc and fasting glucose in patients with T2DM (Table 2). The percentage of patients with low serum miR-96-3p was higher in patients treated with oral hypoglycemic agents than in patients without the treatment; while the percentage of patients with low serum miR-29a-3p and miR-208a-3p was higher in patients treated with insulin than in patients without insulin treatment (Table 3).

### The Effects of GAS5 on Insulin Secretion in INS-1 832/13 Cells

**FIGURE 3:**
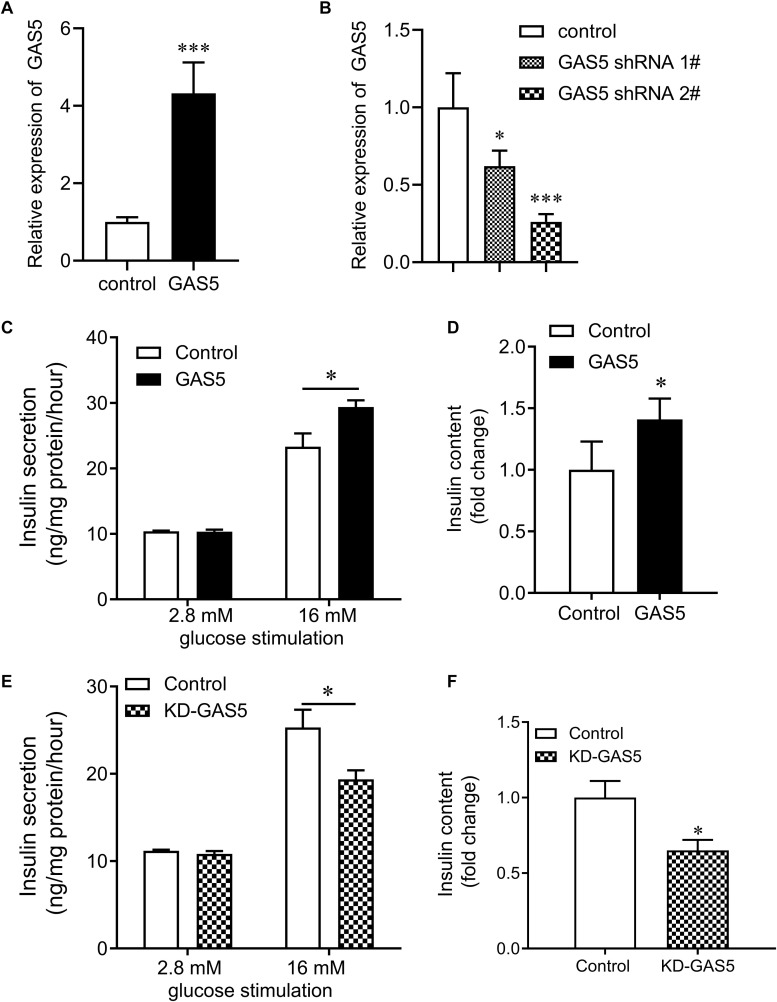
Effects of GAS5 on glucose stimulated insulin secretion in NS-1 832/13 cells. **(A)** QPCR was performed to determine the expression of GAS5 in INS-1 832/13 cells after transfection with lentivirus carrying GAS5 DNA sequence or empty pReceiver-Lv218 vector control. **(B)** QPCR was performed to determine the expression of GAS5 in INS-1 832/13 cells after transfection with lentivirus carrying GAS5 shRNA sequence or scramble control. **(C)** Insulin secretion in GAS5 overexpressed INS-1 832/13 cells after 2.8 mM or 16 mM glucose stimulation. **(D)** Insulin content in INS-1 832/13 cells after GAS5 overexpression. **(E)** Insulin secretion in GAS5 knocked down INS-1 832/13 cells after 2.8 or 16 mM glucose stimulation. **(F)** Insulin content in INS-1 832/13 cells after GAS5 knockdown. KD, knockdown. *p < 0.05.

To investigate whether GAS5 affected insulin secretion in pancreatic β cells, we overexpressed or knocked down (KD) GAS5 in INS-1 832/13 cells by lentivirus transfection. GAS5 expression was significantly increased in the cells infected lentivirus carrying GAS5 DNA sequence compared with the controls (Figure 3A). GAS5 expression was significantly reduced in the cells infected lentivirus carrying GAS5 shRNA sequence compared with the scramble controls (Figure 3B). In addition, the GAS5 shRNA 2# showed a higher efficiency in decreasing GAS5 level compared with the shRNA 1# (Figure 3B). Therefore, shRNA 2# was used for the all next experiments if without specifically referring. We performed MTT assay to determine the cell viability after overexpression and knockdown of GAS5 in INS-1 832/13 cells. We found that GAS5 overexpression significantly increased the cell viability compared with the control, while GAS5 knockdown significantly reduced the cell viability compared with the scramble control (Supplementary Figure S2). Importantly, overexpression of GAS5 increased about 20% of insulin secretion and up to 30% of insulin content compared to negative control (Figures 3C,D). In contrast, knockdown of GAS5 led to significant reduction of glucose-stimulated insulin secretion (30%) and insulin content (40%) compared to negative control (Figures 3E,F). These results suggest that GAS5 has a direct regulatory role on insulin secretion in the β cells.

### GAS5 Interacts With miR-29a-3p, miR-96-3p, and miR-208a-3p and Regulates Their Expression

**FIGURE 4:**
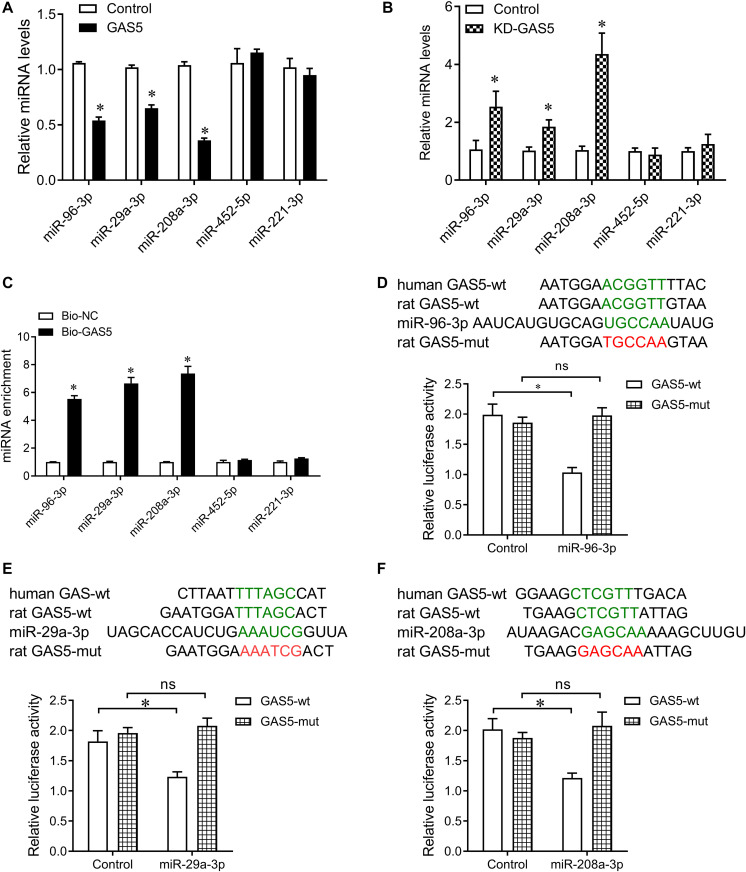
GAS5 negatively regulates the expression of miR-29a-3p, miR-96-3p, and miR-208a-3p. **(A)** QPCR was performed to determine the expression of miR-29a-3p, miR-96-3p, miR-208a-3p, miR-452-5p, and miR-221-3p in INS-1 832/13 cells after transfection with lentivirus carrying GAS5 DNA sequence or empty pReceiver-Lv218 vector control. **(B)** QPCR was performed to determine the expression of miR-29a-3p, miR-96-3p, miR-208a-3p, miR-452-5p, and miR-221-3p in INS-1 832/13 cells after transfection with lentivirus carrying GAS5 shRNA sequence or scramble control. **(C)** Pull-down assay with biotinylated GAS5 probe (Bio-GAS5) was performed to determine the interaction of GAS5 with miR-29a-3p, miR-96-3p, and miR-208a-3p in INS-1 832/13 cells. MiR-29a-3p, miR-96-3p, and miR-208a-3p were significantly enriched in the complex pulled down by Bio-GAS5 compared with Bio-NC. **(D–F)** The wild type binding sites of GAS5 and miRNAs (indicated by green), and the mutant type (indicated by red) were shown (upper). Dual luciferase reporter gene assay was performed to validate the interaction between GAS5 and miR-29a-3p, miR-96-3p, and miR-208a-3p (lower). Data were presented as mean ± SD. *p < 0.05 vs. control. KD, knockdown; ns, no significant.

Bioinformatics analysis was performed to search the potential targeted microRNAs of GAS5 as previous described (Jeggari et al., 2012), which was based on the comprehensive GENCODE gene annotation, including >10000 long non-coding RNA genes and site conservation is evaluated based on 46 vertebrates species, including human and rat. By the bioinformatics analysis, there was conserved binding site for GAS5 on miR-29a-3p, miR-96-3p, and miR-208a-3p in rat and human (Figure 4). Together with the association of GAS5 with miR-29a-3p, miR-96-3p, and miR-208a-3p in serum samples of patients with T2DM (Figure 1B), we then wanted to know whether GAS5 could regulate the expression of miR-29a-3p, miR-96-3p, and miR-208a-3p. We found that overexpression of GAS5 greatly reduced the expression of miR-29a-3p, miR-96-3p, and miR-208a-3p, whereas knockdown of GAS5 significantly increased the expression of miR-29a-3p, miR-96-3p, and miR-208a-3p in INS-1 832/13 cells (Figures 4A,B). However, knockdown or overexpression of GAS5 did not alter the expression of miR-452-5p and miR-221-3p in INS-1 832/13 cells (Figures 4A,B).

Furthermore, we performed pull down assay with biotinylated GAS5 probe to determine the interaction of GAS5 with miR-29a-3p, miR-96-3p, and miR-208a-3p in INS-1 832/13 cells. The pull-down assay results showed that miR-29a-3p, miR-96-3p, and miR-208a-3p significantly enriched in the complex pull down by biotinylated GAS5. In addition, miR-452-5p and miR-221-3p did not enriched in the complex pull down by biotinylated GAS5 (Figure 4C). To validate the interaction of GAS5 with miRNAs, we cloned the binding sequence of rat GAS5 downstream to a luciferase reporter gene (GAS5-wt; indicated by green in Figures 4D–F), and constructed its mutant version (GAS5-mut; indicated by red in Figures 4D–F) by the binding site mutagenesis. The luciferase activity of cells co-transfected rat miR-29a-3p, miR-96-3p, and miR-208a-3p mimics and GAS5-wt was significantly reduced compared to scramble controls. Moreover, miR-29a-3p, miR-96-3p, and miR-208a-3p -mediated repression of luciferase activity was abolished by the mutant binding site (Figures 4D–F). These results show that GAS5 interacts with miR-29a-3p, miR-96-3p, and miR-208a-3p in INS-1 832/13 cells.

**FIGURE 5:**
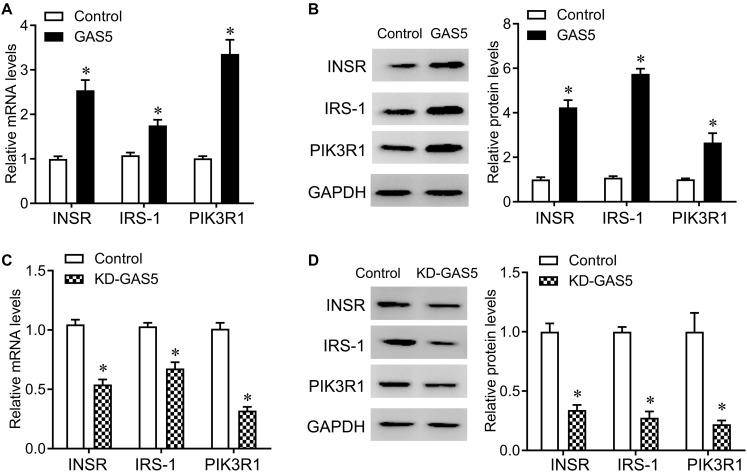
GAS5 positively regulates the expression of INSR, IRS-1 and PIK3R1. **(A,B)** QPCR **(A)** and Western blot **(B)** were performed to determine the mRNA and protein levels of INSR, IRS-1 and PIK3R1 in INS-1 832/13 cells after transfection with lentivirus carrying GAS5 DNA sequence or empty pReceiver-Lv218 vector control. **(C,D)** QPCR **(C)** and Western blot **(D)** were performed to determine the mRNA and protein levels of INSR, IRS-1 and PIK3R1 in INS-1 832/13 cells after transfection with lentivirus carrying GAS5 shRNA sequence or scramble control. The expression was normalized to control. Data were presented as mean ± SD. *p < 0.05 vs. control. KD, knockdown.

Insulin receptor, IRS-1 and PIK3R1 were closely involved in insulin secretion. In addition, by the bioinformatic tool^1^, we also found that miR-96-3p conversely targeted the 3′UTR of INSR, PIK3R1, and IRS1, miR-29a-3p conversely targeted PIK3R1 and IRS1, and miR-208a-3p conversely targeted PIK3R1 among mammals, including human and rat (Data not show). Therefore, we tested the expression of INSR, IRS-1, and PIK3R1 after GAS5 overexpression or knockdown. Knockdown of GAS5 significantly reduced the expression of INSR, IRS-1 and PIK3R1, whereas overexpression of GAS5 greatly upregulated the expression of INSR, IRS-1, and PIK3R1 at mRNA and protein levels in INS-1 832/13 cells (Figure 5).

## Discussion

In this study, we found that the serum levels of GAS5 were significantly decreased in patients with T2DM compared with healthy control subjects. These results were consistent with previous report that decreased serum GAS5 levels were associated with T2DM (Carter et al., 2015). We further demonstrated that overexpression of GAS5 increased glucose-stimulated insulin secretion and insulin content compared to negative control. In contrast, knockdown of GAS5 led to significant reduction of glucose-stimulated insulin secretion and insulin content.

GAS5 plays a widespread role in different diseases, including cancers, inflammation-related diseases, obesity and autoimmune diseases (Ma et al., 2016; Zhou et al., 2019). GAS5 is downregulated in multiple cancers and acts a tumor suppressor in breast cancer, prostate cancer, lung adenocarcinoma, and pancreatic cancer (Lu et al., 2013; Liu et al., 2018). The levels of GAS5 are significantly decreased in pancreatic cancer tissues compared with normal control. Overexpression of GAS5 in pancreatic cancer cells inhibits cell proliferation by negatively regulating cyclin-dependent kinase 6 (Song et al., 2014). In addition, GAS5 participates in the inflammation progress. For example, GAS5 contributes to the pathogenesis of osteoarthritis by acting as a negative regulator of miR-21 and thereby regulating chondrocytes survival (Mayama et al., 2016). The abnormal levels of GAS5 also alter glucocorticoid response in part through modulation of the glucocorticoid receptor transcriptional activity via its decoy RNA “glucocorticoid response element” (Madhyastha et al., 2012; Lucafo et al., 2015). Qi et al. (2017) analyzed the differentially expressed lncRNAs in lymphatic endothelial cells from non-diabetic patients and T2DM patients and found that GAS5 was dysregulated in lymphatic endothelial cells of T2DM patients (Qi et al., 2017), suggesting that GAS5 is involved in the pathogenesis of diabetes-related complications. In addition, knockdown of GAS5 arrests cell cycle G1 and impair insulin synthesis and secretion in Min6 cells, and decreased the expression of insulin gene and transcription factors, Pancreatic And Duodenal Homeobox 1 (PDX1) and MAF BZIP Transcription Factor A (MAFA) in primary isolated islets (Jin et al., 2017). Recently, Esguerra et al. (2020) also find that GAS5 knockdown or dexamethasone treatment resulted in reduced GAS5 levels, leading to concomitant reductions in glucocorticoid receptor (GR), PDX1, NK6 Homeobox 1 (NKX6-1), and synaptotagmin 13 (SYT13) proteins. In line with these results, we found that knockdown of GAS5 led to significant reduction of glucose-stimulated insulin secretion and insulin content in rat pancreatic β cells. These results indicate that GAS5 might perform as a regulator, maintaining β cell identity and function by affecting insulin synthesis and secretion.

The functions of lncRNAs are achieved by interacting with endogenous miRNAs. We demonstrated that decreased serum GAS5 levels were negatively correlated with high serum levels of miR-29a-3p, miR-96-3p, and miR-208a-3p in patients with T2DM. MiR-96-3p is dysregulated during healing of diabetic wounds (Locke et al., 2014) and is important for β-cell function (Chakraborty et al., 2013; Yang et al., 2016). MiR-96-3p is induced strongly by saturated fatty acids in the development of hepatic insulin resistance. Overexpression of miR-96-3p can cause an impairment of insulin signaling and glycogen synthesis in hepatocytes by repressed the expression of INSR and IRS-1 at the post-transcriptional level (Yao et al., 2019).

In addition, miR-29 family has important roles in insulin secretion and insulin resistance. Overexpression of miR-29a/b/c in 3T3-L1 adipocytes could largely repress insulin-stimulated glucose uptake through inhibiting Akt activation and cause insulin resistance (He et al., 2007). MiR-29 expression in human hepatoma cells is controlled by forkhead box A2 (FOXA2), a key gene in hepatic energy homeostasis. In turn, miR-29 fine-tunes FOXA2-mediated activation of key lipid metabolism genes (Kurtz et al., 2014). Global or hepatic insufficiency of miR-29 potently prevents the onset of diet-induced insulin resistance by negatively regulating insulin signaling via PIK3R1 regulation (Dooley et al., 2016), suggesting that miR-29 is an important regulatory factor in metabolism homeostasis (Massart et al., 2017).

MiR-208a-3p was a heart-specific microRNA and was required for cardiomyocyte hypertrophy and fibrosis in response to stress and hypothyroidism (van Rooij et al., 2007). In this study, by the bioinformatic tools (see text footnote 1), we also found that miR-96-3p conversely targeted 3′UTR of INSR, PIK3R1, and IRS1, miR-29a-3p conversely targeted PIK3R1 and IRS1, and miR-208a-3p conversely targeted PIK3R1 among mammals, including human and rat (Data not show). In addition, we found that GAS5 negatively regulated the expression of miR-29a-3p, miR-96-3p, and miR-208a-3p in rat pancreatic β cells. Moreover, GAS5 positively regulated the expression of INSR, PIK3R1 and IRS1 rat pancreatic β cells. Therefore, we supposed that GAS regulated the expression of INSR, PIK3R1 and IRS1 through competitively interacting miR-29a-3p, miR-96-3p, and miR-208a-3p, but we cannot exclude other mechanisms play a role in this regulation, such as epigenetic mechanism (Sun et al., 2017). MiR-452-5p and miR-221-3p are two valid targets of GAS5 in renal tubular cells and mesangial cells, respectively, and both of them are involved in diabetic nephropathy (Ge et al., 2019; Xie et al., 2019). However, the levels of miR-452-5p and miR-221-3p were comparable between high GAS5 group and low GAS5 group. In addition, knockdown or overexpression of GAS5 did not alter the expression of miR-452-5p and miR-221-3p. We supposed that the targets of GAS5 were cell- or tissue-specific.

## Conclusion

We demonstrated that decreased serum GAS5 levels were negatively associated with HbAlc and fasting glucose in patients with T2DM. Overexpression of GAS5 increased glucose-stimulated insulin secretion and insulin content by inhibiting miR-29a-3p, miR-96-3p, and miR-208a-3p. Thus, our findings reveal that GAS5 is a biomarker and novel therapeutic target for T2DM treatment.

## Data Availability Statement

The raw data supporting the conclusions of this article will be made available by the authors, without undue reservation.

## Ethics Statement

The studies involving human participants were reviewed and approved by the Ethics Committee of Xiangya Hospital, Central South University. The patients/participants provided their written informed consent to participate in this study.

## Author Contributions

All authors designed the study, contributed to the writing of the manuscript, and read and approved the final manuscript. RG, JG, and PX performed the data analysis. YL, PX, and JG performed the reverse transcription-quantitative polymerase chain reaction, western blot, and luciferase reporter assay.

## Conflict of Interest

The authors declare that the research was conducted in the absence of any commercial or financial relationships that could be construed as a potential conflict of interest.

## Footnotes

1 http://www.targetscan.org/
