# Supplementary material for: Long Non-coding RNA GAS5 Maintains Insulin Secretion by Regulating Multiple miRNAs in INS-1 832/13 Cells
Source: Front Mol Biosci. 2020 Sep 24;7:559267. doi: 10.3389/fmolb.2020.559267 (PMC7542228; doi:10.3389/fmolb.2020.559267)
Supplement: Supplementary file 1 [file Data_Sheet_1.PDF]

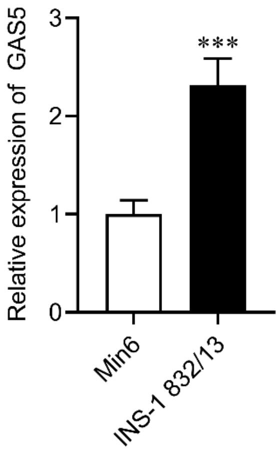

Supplementary Figure 1 The GAS5 expression in Min6 and INS-1 832/13 cells. QPCR was performed to determine the expression of GAS5 in Min6 cells and INS-1 832/13 cells. The relative expression was normalized to Min6 cells. Data were presented as mean ± SD. *** $p < 0.05$ vs. Min6.

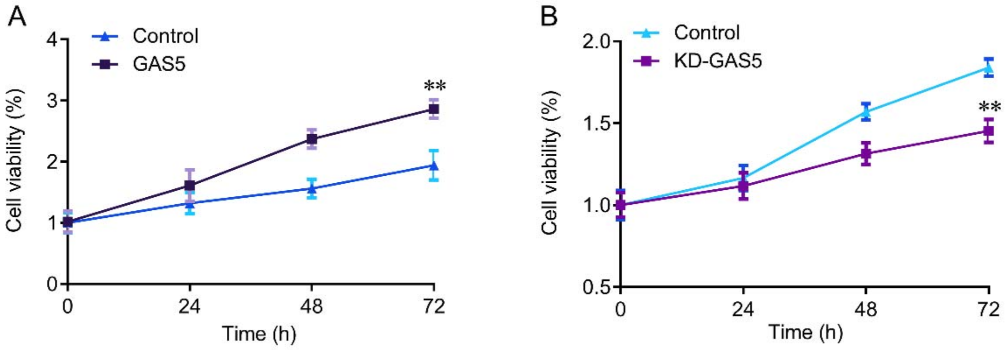

Supplementary Figure 2 Effects of GAS5 on cell viability in NS-1 832/13 cells. (A) MTT was performed to determine the cell viability in INS-1 832/13 cells after transfection with lentivirus carrying GAS5 DNA sequence or empty pReceiver-Lv218 vector control. (B) MTT was performed to determine the cell viability in INS-1 832/13 cells after transfection with lentivirus carrying GAS5 shRNA sequence or scramble control.
